# Periprostatic Adipose Tissue Microenvironment: Metabolic and Hormonal Pathways During Prostate Cancer Progression

**DOI:** 10.3389/fendo.2022.863027

**Published:** 2022-04-13

**Authors:** Paula Alejandra Sacca, Juan Carlos Calvo

**Affiliations:** ^1^ Laboratorio de Química de Proteoglicanos y Matriz Extracelular, Instituto de Biología y Medicina Experimental (IBYME)—CONICET, Buenos Aires, Argentina; ^2^ Departamento de Química Biológica, Facultad de Ciencias Exactas y Naturales, Universidad de Buenos Aires, Buenos Aires, Argentina

**Keywords:** prostate cancer, metabolism, microenvironment, periprostatic adipose tissue, proteomics, secretome, adipose tissue, benign prostatic hyperplasia

## Abstract

The periprostatic adipose tissue (PPAT) is a site of invasion of prostate cancer (PCa) and is part of the microenvironment. It was shown that PPAT secretes factors and fatty acids (FAs) that alter the microenvironment of the PCa. The PPAT secretome of patients with PCa-T3 stage (PPAT-T3) has a metabolic profile enriched in several pathways related to energy production, indicating a greater energy requirement by the tumor, when compared to that of patients in the PCa-T2 stage (PPAT-T2). PPAT-T3 also shows enrichment in pathways related to hormone response, polyamine synthesis, and control of protein synthesis, through amino acid, RNA, and nucleotide metabolism. PPAT-T2 and PPAT-BPH secretomes have less complex metabolic profile, both related with energy balance, while PPAT-BPH has hormone response through insulin pathway. Undoubtedly, a deeper characterization of the human PPAT will lead to a better understanding of the disease and possibly allow new stratification factors and the design of a specific therapy that targets crucial components of the tumor microenvironment as another way to treat or control the disease.

## Introduction

Obesity and metabolic syndrome have been related to many different types of cancer. To our understanding, no integrative work has been performed involving the main metabolic pathways as possible targets for diagnosis and/or treatment. Through proteomic and database analyses from conditioned media obtained from the periprostatic adipose tissue (PPAT) that was surgically removed from patients with prostate cancer (PCa) at different stages of the disease, we have compiled several metabolic and hormone signaling pathways that sound promising to be considered as potential targets.

## Adipose Tissue and Cancer

The adipose tissue is both a metabolically active endocrine organ and an energy depot, which can secrete adipokines and other molecules that contribute to paracrine and autocrine signaling networks in the tumor microenvironment. The tumor microenvironment is critical for cancer development, progression, metastasis, and therapeutic response. It plays a critical role in lipid metabolism, insulin sensitivity, inflammation, energy balance, angiogenesis, and cell proliferation. The adipose tissue, as a whole and its cells’ components, participates in the metabolic interplay with neoplastic cells. They dynamically adapt to the metabolic needs of cancer cells, thus participating in tumorigenesis and resistance to treatments. However, the underlying mechanisms of adipose-tissue-induced cancer initiation and progression are still unclear and controversial.

## The Periprostatic Adipose Tissue: Key Contributor of Tumor Microenvironment

PPAT is a site of invasion of PCa and part of the microenvironment. It was shown that PPAT secretes protein factors and fatty acids (FAs) (1–8) that alter the microenvironment of the PCa.

## Prostate Cancer Metabolic Changes

The metabolism of the prostate cells exhibits unique and distinct profiles during the different stages of the disease that leads to the progression and metastasis of PCa (9). PCa cells do not obey the classical Warburg effect phenotype, as seen in other solid tumors, where the malignant cells change their dominant route of ATP production by oxidative phosphorylation to aerobic glycolysis. In the early phases of PCa malignant transformation and tumor progression, both rely on lipids and other energetic molecules for energy and not on aerobic respiration. Alterations in lipid and FA metabolism, which are necessary for energy production, membrane synthesis, and post-translational modification of signaling molecules, are increasingly being recognized as vital in these phases (10). In PCa, a malignant metabolic shift occurs, re-establishing an intact tricarboxylic acid (TCA) cycle and converting prostate cells from citrate-producing to citrate-oxidizing cells, thereby enhancing glucose metabolism (11). Through this phenotypical, more energetically favorable metabolic switch, citrate is used for oxidative phosphorylation and biosynthetic processes such as lipogenesis. It is only in the late stage, with numerous mutation events, that PCa will begin to exhibit the Warburg effect and have a higher rate of glucose uptake. By contrast, normal prostate epithelial cells exhibit a truncated TCA cycle to enable production of citrate, a key component of prostatic fluid, resulting in high rates of glycolysis (12).

## Metabolism Dysfunction of Periprostatic Adipose Tissue in Prostate Cancer

Recently, we reported the secretome of PPAT from patients with PCa (T2 and T3 stage) and with BPH (4) using a proteomic approach. We found that locomotion process and extracellular matrix, structural proteins and immune response function were differentially regulated according to the PCa stage, while catalytic activity, reproduction process, metabolism, and energy pathways were in PPAT-T3. We detected enriched metabolic pathways related to energy balance, hormone response, and control of protein synthesis. Genes related with those pathways are shown in **Table 1**. Energy pathways associated with the PPAT-T3 secretome were lipid and lipoprotein metabolism, carbohydrates and glucose metabolism, glycolysis, gluconeogenesis, regulation of ornithine decarboxylase (ODC), advanced glycosylation of end products, receptor signaling, TCA cycle variation III, GDP-glucose biosynthesis, and pyruvate fermentation to lactate. Pathways related to hormonal response were insulin/IGF1/class phosphoinositide 3-kinase (PI3K) signaling events mediated by protein kinase B (Akt) pathways and hormone-sensitive lipase (HSL)-mediated triacylglycerol hydrolysis. Among the pathways related to protein synthesis were RNA/mRNA, nucleotides, amino acids and derivatives metabolism, and aspartate degradation II. PPAT-T2 secretome showed a metabolic profile with less complexity and lesser energy requirements than in advanced disease. Biological enriched pathways related to energy balance were lipid digestion, mobilization and transport, lipoprotein metabolism, and glycolysis, a well-established feature of cancer. The secretome of PPAT in BPH patients was related to hormone response through the insulin pathway, which was previously reported to be associated with prostate size (13). In addition, the secretome profile of PPAT in patients with BPH showed the metabolic pathways related to energy production through metabolism of lipids and lipoproteins, lipid digestion, mobilization, and transport; all necessary for cell division.

**Table 1:** Detected genes in the prostate tumor microenvironment associated with the enriched metabolic pathways.

Metabolic Pathways	Genes Names
**PPAT-T3**	
Glucose metabolism	GOT1 GPI MDH1 MDH2 PGK1 PRKACB PYGL TPI1 UGP2
Glycolysis	GPI PGK1 TPI1
Gluconeogenesis	GOT1 GPI MDH1 MDH2 PGK1 PRKACB TPI1
GDP-glucose biosynthesis/glucose and	PGM1 PGM5
glucose-1-phosphate degradation	
TCA cycle variation III (eukaryotic)	ACO1 MDH1 MDH2
pyruvate fermentation to lactate	LDHA LDHB
Metabolism of lipids and lipoproteins	A2M ACSL1 APOA1 APOB APOC3 ECI1 FABP4 GC GPD1 HSPG2 IDH1 LTA4H
Lipoprotein metabolism	A2M APOA1 APOB APOC3 HSPG2 P4HB
Lipid digestion, mobilization, and transport	A2M APOA1 APOB APOC3 FABP4 HSPG2 P4HB PLIN1 PRKACB
Hormone-sensitive lipase (HSL)-mediated	FABP4 PLIN1 PRKACB
triacylglycerol hydrolysis	
Metabolism of carbohydrates	GOT1 GPI HK1 MDH1 MDH2 PGD PGK1 PRKACB PYGL SLC2A1 TPI1 UGP2
Metabolism of RNA/mRNA	ELAVL1 HSPA1A HSPA1B HSPB1 PSMA1 PSMA5 PSMA6 PSMA7 PSMB1 PSMB4
Metabolism of nucleotides	CAT GLRX HPRT1 PNP TXN
Regulation of ornithine decarboxylase (ODC)	PSMA1 PSMA5 PSMA6 PSMA7 PSMB1 PSMB4 PSMB5
Advanced glycosylation end product receptor signaling	CAPZA2 PRKCSH S100B
Metabolism of amino acids and derivatives	FAH GOT1 PSMA1 PSMA5 PSMA6 PSMA7 PSMB1 PSMB4 PSMB5 QDPR
aspartate degradation II	GOT1 MDH1 MDH2
Insulin pathway/IGF1 pathway/class I PI3K	A2M ACTN1 ACTN4 ACTR3 ARPC5 B2M CAT CLTC COL1A1 COL1A2 CP DSP EEF2
signaling events mediated by Akt	ELANE ENO1 FABP4 FGA FGB FGG FN1 GDI1 GSN HK1 HNRNPA1 HSPA1A HSPA1B
	HSPA4 HSPB1 IGF2BP1 IQGAP1 IRS2 JUP LAMC1 LDHA LGALS1 LRP1 MMP9 NCAM1
	NCL NPM1 PEBP1 PGK1 PGM1 PKM PLG PRDX1 PRDX3 PRKACB PTPN11 SERPINE1
	SLC2A1 SORBS1 SOS1 SPTBN1 TF TLN1 TXN USO1 VASP VCL VTN YWHAG YWHAZ
**PPAT-T2**	
Glycolysis	GPI TPI1
Lipoprotein metabolism	A2M APOA1 APOA2
Lipid digestion, mobilization, and transport	A2M APOA1 APOA2 FABP4
**PPAT-BPH**	
Insulin Pathway/IGF1 pathway/Class I PI3K	A2M ACTN1 ACTN4 FABP4 FGA FGB FN1 JUP LGALS1 PGM1 PRDX1 SERPINE1 TF
Lipoprotein metabolism	A2M APOA1 APOA2
Lipid digestion, mobilization, and transport	A2M APOA1 APOA2 FABP4

PPAT-T3, PPAT periprostatic adipose tissue of patients with prostate cancer T3 stage; PPAT-T2, periprostatic adipose tissue of patients with prostate cancer stage T2; PPAT-BPH, periprostatic adipose tissue of patients with benign prostatic hyperplasia.

Regarding metabolic processes, only patients with PCa in T3 stage showed an increase in the metabolic process involving NADP and pigment when compared to PCa-T2 stage. In the NADP metabolic process, phosphogluconate dehydrogenase (decarboxylating) (PGD) and isocitrate dehydrogenase (NADP) cytoplasmic (IDH1) proteins were expressed only in advanced disease. Regarding the metabolism involved in pigment processing, delta-aminolevulinic acid dehydratase (ALAD) and hypoxanthine phosphoribosyltransferase-1 (HPRT1) were detected in the same PCa stage. In addition, glycosylation was present as one of the altered metabolic processes in PCa T3 stage with the involvement of UDP-glucose:glycoprotein glucosyltransferase 1 isoform 2 (UGGT1). When we compared the metabolic processes between BPH patients and PCa-T2 stage, BPH showed an increase in NADP and pigment metabolisms through transketolase (TKT) and hemopexin, respectively, while patients with PCa-T2 stage had an exacerbated secondary metabolic process through the aldo-keto reductase family 1 member C3 (AKR1C3).

Tumor cells must adapt to survive, both satisfying their biomass and need for fuel in an unfriendly microenvironment and, thus, switch their metabolic functions according to microenvironment evolution. The interaction between the microvesicles released by the tumor and stromal cells/adipose stem cells into the microenvironment plays a fundamental role in facilitating the progression of the cancer (14, 15). It is known that the microenvironment of primary tumor and metastatic niches differ in their cellular or acellular components, available nutrients, and metabolites released. In addition, PCa cells could take secreted metabolites produced in the PPAT microenvironment by diffusion and get them through physical crosstalk with adjacent cell components of this depot or capture extracellular vesicles released by neighboring cells. Thus, metabolic remodeling is not only restricted to tumor cells but also generated in the environment and in non-tumor cells, resulting in a feedback loop, where microenvironmental cells drive metabolic changes in tumor cells, thus providing essential metabolic resources for tumor growth. As we previously reported, proteins found in the PPAT secretome could derive not only from PPAT but also from the tumor and further packaged into exosomes, as some proteins have not been reported in the adipose tissue. We found that the exosome fraction was the most represented cellular component in the PPAT secretome, with 209 proteins for CM-T3, 39 proteins for CM-T2, and 34 proteins for CM-BPH, showing that the profile of protein content changed in response to the microenvironment during progression of the tumor and with the androgenic context (4). Considering the relevance that AR signaling axis is being given as a focus for prostate cancer therapy, it is key to understand how this axis alters the protein cargo and secretion on exosomes found in the PPAT microenvironment.

Epithelial cancer cells induce the Warburg effect (aerobic glycolysis) in neighboring stromal fibroblasts. These cancer-associated fibroblasts secrete lactate, which is converted to pyruvate and utilized in the mitochondria by the better oxygenated tumor cells (energy metabolites resulting from aerobic glycolysis). Epithelial cancer cells could then take up these energy-rich metabolites and use them in the mitochondrial TCA cycle, thereby promoting efficient energy production (ATP generation *via* oxidative phosphorylation), resulting in a higher proliferative capacity, giving place to the so-called reverse Warburg effect (16). Cancer cells that grow in the presence of stromal cells, for example, adipocytes, preadipocytes, or fibroblasts, will adapt their metabolism (oxidative phosphorylation and β-oxidation) to take full advantage of the metabolites (lactate, ketones, glutamine, and FAs) provided by the local host cells (17). Lactate not only participates in metabolic processes but also contributes to oncogenic signaling pathways.

Previous reports showed that PPATs have a different FAs composition according to the pathological stage (8, 18), which results in a different/altered lipid metabolism. We could observe in PPAT-T3 secretome that the metabolism of lipids is enriched. FA incorporation into oxidative pathways is reduced in aggressive human cancer cells and, instead, shunted into pathways to generate structural and signaling lipids. Cancer cells do not solely rely on *de novo* lipogenesis but also utilize exogenous FAs to generate lipids required for proliferation and pro-tumorigenic signaling. It has been observed that palmitic acid incorporation into complex lipids is increased in aggressive cancer cells into glycerophospholipid, sphingolipid, and ether lipid pathways (19). Previously, we reported the presence of palmitic acid in PPAT of patients with tumor, and it was significantly increased when compared to BPH (8). A study demonstrated that specific lipid translocation takes place between human-derived adipocytes and PC3 cells (20). The supply of FAs from adipocytes around cancer cells is conducive to cancer growth and progression. Recently, it was reported that PCa uses extracellular FAs as both fuel for oxidation and as primary substrates for complex lipid synthesis such as triacylglycerols (TAGs) and that the higher availability of extracellular lipids further enhances FA flux in these cells. In addition, the heterogeneity of lipid metabolism was observed in several PCa cell lines and the potential role that obesity-associated dyslipidemia or the host-circulating lipidome have on PCa progression (21). A high concentration of palmitate and glucose *in vitro* induces cell proliferation, migration, and oxidative stress in PC3 cells. Palmitate presents a rapid and initial effect, while a glucose-rich environment stimulates cells later on, maintaining high levels of cell proliferation (22). Alterations in lipid and, particularly, FA metabolism, necessary for energy production, membrane synthesis, and post-translational modification of signaling molecules, are being recognized as vital for the early phases of malignant transformation and in tumor progression (11). Enhanced synthesis and uptake are hallmarks of PCa cells and are androgen regulated (23). Very recently, it was shown that in human malignant PCa tissue, the increased uptake of FAs was mediated by upregulation of the fatty acid translocase CD36. In PCa mouse and human preclinical models, silencing of CD36 reduced FAs and cell proliferation and PCa severity (24). Previously, we reported the presence of CD36 in PPAT-T3 secretome (4). Thus, inhibition of FAs uptake and/or key lipid metabolism pathways could be a promising alternative to treat PCa. Shrihari et al. (9) identified at least two subgroups of PCa patients who exhibited significant poor prognosis (30%–40% relapse within the first 72 months relative to the best prognosis cluster that showed <20% relapse even by 120 months), which showed considerable deregulation of pathways involved in synthesis and catabolism of complex forms of lipids and carbohydrates, and these were exhibited in parallel or within glycolysis, a common form of energy production in cancer cells.

The role of glucose metabolism and PCa has not been well elucidated yet. While glucose uptake does not appear to be increased early in PCa cells, it does appear to be involved in the progression of the disease and cellular division (12). PCa is often not glycolytic. It was shown that a glycolytic switch occurred when PCa cancer cells were co-cultivated with adipocytes due to a twofold increase in lactate in this condition (25). Glycolysis is an altered pathway in the metabolism of PCa cells (11). We found that PPAT microenvironment in a more advanced stage (T3) showed to be enriched in gluconeogenesis, glycolysis, and pyruvate fermentation to lactate, while in a T2 stage, glycolysis was enriched. Recently, it was shown that microvesicles released by tumors mediated glycometabolic reprogramming of stromal cells in oral squamous cell carcinoma. Normal human gingival fibroblasts, primed with the tumor microvesicles, exhibited a phenotype switch to cancer-associated fibroblasts (CAF) and underwent a degradation of caveolin-1 (CAV1). CAV1 degradation further induced the metabolic switch to aerobic glycolysis in the fibroblasts. The microvesicle-activated fibroblasts absorbed more glucose and produced more lactate, resulting in a mechanism for tumor progression by a crosstalk between tumor and stromal cells through the reverse Warburg effect (15). This situation could be assimilated to PCa-T3 stage, where PPAT cell components, like adipose stem cell (ASC) and fibroblasts, are primed with PCa microvesicles. PCa cell microenvironment subverts PCa patient adipose stem cells to undergo neoplastic transformation when primed with PCa cell conditioned media (14). Previous reports showed that oncogene-associated metabolic signatures in PCa support the notion that PI3K activation generally results in a glycolytic phenotype, whereas MYC induces aberrant lipid metabolism, with substantial heterogeneity (11). PI3K and AR pathways are key targets in PCa, as they seem to be reciprocally regulated. Comparing both pathways, it seems feasible to target PI3K due to its dominant role over AR signaling. While activation of PI3K pathway is usually associated with resistance to androgen deprivation therapy, its inhibition is antiproliferative, whereas an increase in AR signaling has proliferative effects. An overactivation of the PI3K axis was seen in a subset of primary PCa and castration-resistant prostate cancer (CRPCa), which has phosphatase and tensin homolog (PTEN)-deficient or PI3K-activating mutations. In addition, the activation of PI3K/Akt is critical for the induction of epithelial–mesenchymal transition (EMT) by growth factors, including insulin-like growth factor 1 (IGF-1), thus pinpointing this kinase as an interesting target of consideration.

Regarding the NADP metabolic process, as detected in our reported secretome, the 6PGD enzyme is critically important for the pentose phosphate pathway (PPP). 6PGD was very recently reported to enhance the stability of androgen receptor (AR) protein, revealing a positive feedback loop between androgen signaling and the PPP, which enhances growth and survival of tumor cells. Suppression of 6PGD decreased lipogenesis and RNA biosynthesis and elevated reactive oxygen species (ROS) levels in cancer cells attenuating cell proliferation and tumor growth. Thus, hormones and glucose metabolism in PCa would drive to a new therapeutic target (26).

Another metabolic pathway of the PPAT-T3 secretome that emerged from the analysis was the regulation of ornithine decarboxylase (ODC). ODC is the rate-limiting enzyme of polyamine synthesis. One of the first events in cell proliferation is the induction of polyamine biosynthesis, and it is known that overexpression of ODC, beyond a certain minimum threshold, can induce cell transformation and tumor promotion. In hyperplasic diseases, there is an increase in the activity of the ODC enzyme, while polyamine pathway is altered in stages as early as high-grade prostatic intraepithelial neoplasia. The prostate presents high levels of polyamines, and they increase in tumor tissues. In addition, it was shown that the inhibition of polyamine metabolism enzymes is associated with increased adipose tissue and weight gain in human and animal models (27). Alpha-difluoromethylornithine, an inhibitor of ODC, depleted the cellular polyamines and prevented triglyceride accumulation and differentiation in 3T3-L1 cells (28). In PCa cells, *ODC* and androgen receptor are mutually regulated. Overexpression of the ODC protein causes malignant transformation through activation of the androgen receptor axis, which, in turn, can affect different pathways involved in cell proliferation, cell survival, and tumor invasion (29). Recently, an analysis of *in vivo* metastases and clinical data from PCa patients supports that the PGC-1α (transcriptional co-activator peroxisome proliferator-activated receptor gamma coactivator 1-alpha)/c-MYC/ODC1 axis regulates polyamine biosynthesis and PCa aggressiveness (30).

Using a multiplatform (NMR + LC-MS) metabolomics approach on serum samples to study preoperative metabolic alterations associated with PCa recurrence showed significant alterations in metabolic pathways, including amino acid metabolism, purine and pyrimidine synthesis, tricarboxylic acid cycle, tryptophan catabolism, glucose, and lactate (31), similar to what we detected in the PPAT secretome in high-grade PCa patients.

Although our previous findings pointed toward alterations in metabolic pathways related to energy balance and their possible relation with tumor maintenance and progression, we must not forget the probable outcome of cancer disease: cachexia syndrome. Even with an unaltered caloric intake, there is muscle and fat storage usage that will mobilize lipids and amino acids directly or regulate glucose/glycogen metabolism (both hepatic and muscular). Cachexia will also affect hormone/growth factor pathways, related to adipose tissue metabolism such as insulin or IGF-1 (as observed in our study), and cause a shift towards beige or brown adipose tissue in other types of cancers (32), uncoupling oxidative phosphorylation from the respiratory chain. Although the patients involved in this study did not show signs of cachexia, it is well known that it can no longer be considered a consequence of end-stage tumors. Even more, tissue wasting can be observed at very early stages of tumor development, even before being detectable (30). In relation to this, using combined analysis of serum and plasma samples, with diverse metabolomic platforms, it was possible to discriminate between PCa and BPH, thus indicating that amino acid metabolism could be a marker for PCa when compared to BPH (33).

A recent review by Xu et al. (34) specifically pinpoints to fatty acid metabolism with regard to the possible connection between prostate cancer and obesity, indicating that steps involved in this metabolism could be targets for PCa diagnosis and treatment. Here, we presented a broader view that involves carbohydrate, lipid, and protein metabolisms with connection to hormone signaling pathways.

## Discussion

There are key differences in PPAT metabolic behavior during PCa onset and progression. PPAT-T3 secretome has a metabolic profile enriched in several biological pathways related to energy balance, probably indicating a greater energy requirement by the tumor when compared to PPAT-T2. In addition, in tumors with high pathological grade, the secretome of PPAT is enriched in pathways related to hormone response, polyamine synthesis (through regulation of ornithine decarboxylase), and control of protein synthesis, like amino acid, RNA, and nucleotide metabolism, whose catabolic products could be required for tumor growth. PPAT-T2 and PPAT-BPH secretome showed a lesser complex metabolic profile. While PPAT-T2 secretome showed biological pathways related with energy balance, PPAT-BPH secretome was related to both energy balance and hormone response through insulin pathway. Thus, the knowledge of the metabolic profile of PPAT microenvironment could be a useful indicator for early diagnosis of high-risk tumors. We suggest that exploring the metabolic reprogramming, also related with energy interchange, has both therapeutic and diagnostic implications. The development of therapeutic metabolic approaches must, therefore, consider that the metabolic reprogramming of tumor cells evolves with microenvironmental changes. Therefore, combined therapies that consider these metabolic changes of the disease to attack tumor and target cancer microenvironment should be developed.

## Author Contributions

PAS proposed the concept for the review. PAS and JCC contributed to writing the paper. All authors contributed to the article and approved the submitted version.

## Funding

This study was supported by the Argentine National Cancer Institute (2014-2016 11360588) and a generous donation from Fundación Honorio Bigand.

## Conflict of Interest

The authors declare that the research was conducted in the absence of any commercial or financial relationships that could be construed as a potential conflict of interest.

## Publisher’s Note

All claims expressed in this article are solely those of the authors and do not necessarily represent those of their affiliated organizations, or those of the publisher, the editors and the reviewers. Any product that may be evaluated in this article, or claim that may be made by its manufacturer, is not guaranteed or endorsed by the publisher.
